# Biological Activities and Chemical Composition of Methanolic Extracts of Selected Autochthonous Microalgae Strains from the Red Sea

**DOI:** 10.3390/md13063531

**Published:** 2015-06-03

**Authors:** Hugo Pereira, Luísa Custódio, Maria João Rodrigues, Carolina Bruno de Sousa, Marta Oliveira, Luísa Barreira, Nuno da Rosa Neng, José Manuel Florêncio Nogueira, Salman A. Alrokayan, Fouzi Mouffouk, Khalid M. Abu-Salah, Radhouan Ben-Hamadou, João Varela

**Affiliations:** 1 Centre of Marine Sciences, University of Algarve, Faro 8005-139, Portugal; E-Mails: hgpereira@ualg.pt (H.P.); lcustodio@ualg.pt (L.C.); mary_p@sapo.pt (M.J.R.); carolbrunos@yahoo.com (C.B.S.); mmfoliveira@live.com.pt (M.O.); lbarreir@ualg.pt (L.B.); 2 Department of Chemistry and Biochemistry and Center of Chemistry and Biochemistry, Faculty of Sciences, University of Lisbon, Campo Grande, Ed. C8, Lisbon 1749-016, Portugal; E-Mails: nunoneng@gmail.com (N.R.N.); jmnogueira@fc.ul.pt (J.M.F.N.); 3 King Abdullah Institute for Nanotechnology, King Saud University, Riyadh 11451, Saudi Arabia; E-Mails: salrokayan@ksu.edu.sa (S.A.A.); fmouffouk@gmail.com (F.M.); 4 Department of Chemistry, Faculty of Science, Kuwait University, Safat 13060, Kuwait; 5 King Abdullah International Medical Research Center, King Abdulaziz Medical City, Riyadh 11426, Saudi Arabia; 6 Department of Biological and Environmental Sciences, College of Arts and Sciences, Qatar University, Doha, Qatar; E-Mail: benhamadou@qu.edu.qa

**Keywords:** antioxidants, bioprospection, BuChE inhibitors, carotenoids, microalgae, oxidative stress, phenolics

## Abstract

Four lipid-rich microalgal species from the Red Sea belonging to three different genera (*Nannochloris*, *Picochlorum* and *Desmochloris*), previously isolated as novel biodiesel feedstocks, were bioprospected for high-value, bioactive molecules. Methanol extracts were thus prepared from freeze-dried biomass and screened for different biological activities. *Nannochloris* sp. SBL1 and *Desmochloris* sp. SBL3 had the highest radical scavenging activity against 1,1-diphenyl-2-picrylhydrazyl, and the best copper and iron chelating activities. All species had potent butyrylcholinesterase inhibitory activity (>50%) and mildly inhibited tyrosinase. *Picochlorum* sp. SBL2 and *Nannochloris* sp. SBL4 extracts significantly reduced the viability of tumoral (HepG2 and HeLa) cells with lower toxicity against the non-tumoral murine stromal (S17) cells. *Nannochloris* sp. SBL1 significantly reduced the viability of *Leishmania infantum* down to 62% (250 µg/mL). *Picochlorum* sp. SBL2 had the highest total phenolic content, the major phenolic compounds identified being salicylic, coumaric and gallic acids. Neoxanthin, violaxanthin, zeaxanthin, lutein and β-carotene were identified in the extracts of all strains, while canthaxanthin was only identified in *Picochlorum* sp. SBL2. Taken together, these results strongly suggest that the microalgae included in this work could be used as sources of added-value products that could be used to upgrade the final biomass value.

## 1. Introduction

Microalgae are found in almost all environments (both aquatic and terrestrial), and it has been suggested that their number may be as high as 50,000 species [1]. This biodiversity and distribution has provided a wide array of biochemicals, some of them enabling microalgae to thrive in niche and extreme habitats [2], while displaying several important bioactivities. Microalgae are thus considered as a promising feedstock for the extraction of secondary metabolites for successful commercial applications (e.g., Martek and BASF/Betatene Ltd.). Several secondary metabolites identified in microalgae have high commercial value and include carotenoids (e.g., astaxanthin, lutein and β-carotene) and long chain polyunsaturated fatty acids (PUFA), such as eicosapentaenoic (EPA) and docosahexaenoic (DHA) acids [3]. The main advantage of microalgae as sources of novel bioactive molecules is their vast biodiversity. Moreover, microalgae are usually fast-growing unicellular organisms that can be cultivated in large-scale systems (e.g., open ponds and photobioreactors), allowing a continuous supply of large quantities of biomass and of desired molecules [4]. Lastly, bulk microalgal biomass or fractions thereof can be used in nutraceutical applications, simultaneously upgrading the total biomass value and limiting the costs associated with the isolation of specific compounds [5].

The interest in microalgae as novel sources of high-value chemicals and/or other products has recently increased due to the efforts of using these organisms as renewable biofuel feedstock [3]. In fact, microalgal biomass is currently considered as one of the most promising feedstocks for the large-scale production of biofuels [6]. However, it has been proposed that commercial biofuel production can only be economically feasible if high-value components of the algal biomass are exploited as co-products, together with the use of the triacylglycerols for the production of biodiesel in a biorefinery setting [6,7,8].

In a previous work, Pereira *et al.* [9] identified and isolated four microalgal strains from environmental water samples collected off Al-Lith in the Red Sea (west coast of Saudi Arabia) by fluorescent-activated cell sorting (FACS). The selected isolates were identified by ribosomal DNA sequencing and classified as chlorophytes belonging to three different genera, namely *Picochlorum*, *Nannochloris* and *Desmochloris*. All strains had inner cell lipid contents ranging from 20% to 25% of the biomass dry weight (DW), with fatty acid profiles appropriate for biodiesel production [9]. In this work, a bioprospection for commercially-relevant metabolites with the biomass of the aforementioned microalgae was performed. These include pigments and secondary metabolites with valuable biological activities (e.g., free radical scavenging, metal chelating and cholinesterase inhibitory activities, as well as cytotoxicity towards human tumoral cell lines and *Leishmania* parasites), which can have a wide application in the food, feed and pharmaceutical industries. To the authors’ knowledge, the biological activities here described have never been reported in *Nannochloris*, *Picochlorum* and *Desmochloris* microalgae.

## 2. Results and Discussion

### 2.1. Antioxidant Activity

Free radicals, more specifically reactive oxygen species (ROS) and reactive nitrogen species (RNS), have both beneficial and deleterious roles in the human body. When present at very low concentrations, they may act as a second messenger in some of the signal transduction pathways [10]. However, when the production of ROS and/or RNS overcomes the antioxidant defenses of the organism, oxidative stress may occur, which is implicated in the pathogenesis of several chronic diseases. The use of antioxidants can thus prevent and/or reduce the severity of those oxidative stress-related diseases, such as cancer, diabetes, cardiovascular disorders and neurological ailments [11,12,13,14,15]. ROS are constantly produced in the brain by excitatory amino acids and neurotransmitters and can lead to oxidative stress with the associated damage to glial and neuronal cells [16]. In this context, the use of antioxidants to prevent cerebral oxidative stress and neuronal loss has gained increasing importance due to their capacity to neutralize free radicals [13,16].

**Figure 1:**
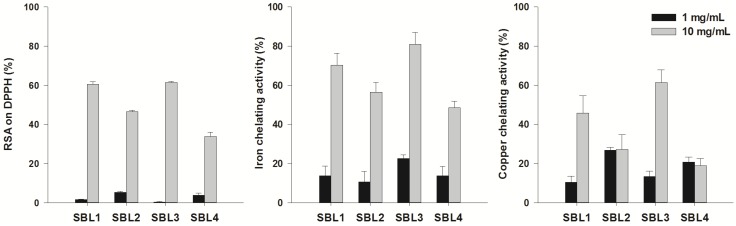
Radical scavenging activity (RSA) on the free radical 1,1-diphenyl-2-picrylhydrazyl (DPPH) and iron and copper chelating activities of methanol extracts of four strains belonging to the *Nannochloris* (SBL1 and SBL4), *Picochlorum* (SBL2) and *Desmochloris* (SBL3) genera. Solid and error bars represent the average and standard deviation values, respectively (*n* = 6). Butylated hydroxytoluene (BHT; positive control) had an RSA of 88% at 1 mg/mL. Ethylenediamine tetraacetic acid (EDTA; positive control) had a metal chelating activity of 76% (copper) and 96% (iron) at a concentration of 1 mg/mL.

In this study, methanol extracts were prepared from dried biomass of four microalgae strains and evaluated for radical scavenging activity (RSA) on the 1,1-diphenyl-2-picrylhydrazyl (DPPH) radical. All species had moderate or high RSA, and the highest values were observed in *Nannochloris* sp. SBL1 and *Desmochloris* sp. SBL3 with RSA values of 60% and 61%, respectively, at a concentration of 10 mg/mL (Figure 1). Butylated hydroxytoluene (BHT; positive control) had an RSA of 88% at a concentration of 1 mg/mL. These results suggest that those species may be sources of compounds with anti-radical properties. Generally, extracts had a higher ability to chelate Fe^2+^ than Cu^2+^ (Figure 1), similar to previous findings in other microalgae [17], such as *N. oculata*. Regarding iron chelation, the highest activity was obtained with *Desmochloris* sp. SBL3 (81%) followed by *Nannochloris* sp. SBL1 (70%) at a concentration of 10 mg/mL (Figure 1). Those species were also able to chelate copper, with values of 61% and 45% at a concentration of 10 mg/mL (Figure 1). Iron may promote the deposition of β-amyloid plaques, which is one of the hallmarks of the progression of Alzheimer’s disease (AD). Moreover, the accumulation of both Fe^2+^ and Cu^2+^ increase the production of ROS through the promotion of the Haber–Weiss/Fenton reaction, which may be responsible for the increase in global oxidative stress parameters observed in AD patients [15,18]. Thus, the use of Fe^2+^ and Cu^2+^ chelators is a valuable strategy in the management of oxidative stress-related neurological disorders [15]. The importance of novel metal chelators is highlighted by the side effects caused by current chelation therapies, which may cause allergic reactions, as well as ophthalmological, auditory and bone toxicity, most probably caused by their lack of specificity or “over-chelation” [19]. Our results suggest that the extract showing higher selectivity for iron corresponds to that of *Picochlorum* sp. SBL2. Lower selectivity between iron and copper chelation is apparent for the *Desmochloris* sp. SBL3 (Figure 1). Interestingly, a recent report on metal chelators suggests that compounds with lower sequestration capacity, but higher specificity may be more promising candidates for novel therapeutic leads. Selective chelators with lower binding activity may be able not only to remove the metal from disease-causing “sinks”, but also to more readily release the metal in other cellular compartments where they are needed [20]. Such mechanism could be a way forward to reduce the adverse effects of known metal chelators.

### 2.2. Acetylcholinesterase, Butyrylcholinesterase and Tyrosinase Inhibitory Activity

AD is characterized by the loss of cholinergic neurons in the forebrain and by a progressive decline in the levels of acetylcholine (ACh) due to hydrolytic reactions catalyzed by acetylcholinesterase (AChE) and butyrylcholinesterase (BuChE) [21]. Thus, research has focused on the identification of cholinergic inhibitors (ChEIs) able to increase the activity of surviving cholinergic neurons in patients with AD. This is the case of the drugs currently used as therapeutics in AD, specifically galantamine/Razadyne^®^, Janssen, an AChE inhibitor, and rivastigmine/Exelon^®^, Novartis, a compound with a dual effect on AChE and BuChE.

In this work, the inhibitory activity (%) towards AChE and BuChE was classified as potent (>50%), moderate (30%–50%), low (<30%) or nil (<5%) [22]. According to this classification, extracts had low and nil inhibitory potential on AChE, but displayed potent activity towards BuChE. The opposite was observed in other microalgae, namely *Botryococcus braunii* and *Nannochloropsis oculata* [23], where a high AChE inhibition was observed, but no relevant BuChE inhibitory activity was detected. The highest BuChE inhibitory effect was obtained with *Picochlorum* sp. SBL2 at the highest concentration tested (10 mg/mL, 69.3% of inhibition; Table 1). Although some of the functions of BuChE are common to AChE (*i.e.*, to catalyze ACh hydrolysis), the exact role of the former enzyme is still unclear [24]. However, there is evidence that some cholinergic neurons contain BuChE instead of AChE [25], and thus, the increase of the cholinergic function through the inhibition of BuChE may be of clinical value. Our results suggest that the microalgae under study contain compounds that can inhibit BuChE, which could be used in combination with AChE inhibitors (Table 1) [26]. Indeed, clinical studies with the dual ChEI, rivastigmine, support a role for the central inhibition of BuChE in addition to AChE in AD therapy [27]. Noteworthy is the fact that BuChE inhibition was evident with the lowest concentration tested and that increasing a concentration applied was not followed by an increase of the inhibitory activity. A possible explanation for this may be related to the balance between inhibitors and activators of BuChE, which can be present in crude extracts [28]. Hence, upon increasing a concentration of the extracts, both types of compounds will equally increase, resulting in an unaltered inhibitory effect.

The extracts were also evaluated for their inhibitory potential against tyrosinase (TYRO), a multifunctional copper-containing enzyme that plays a pivotal role in melanin biosynthesis [29]. TYRO is also involved in neuromelanin formation in the human brain and, due to its oxidase activity, can potentially accelerate the induction of catecholamine quinone derivatives, contributing to dopamine neurotoxicity and to neurodegeneration associated with Parkinson’s disease (PD) [29]. In this sense, TYRO inhibitors have become an attractive target for the treatment of PD. Except for *Nannochloris* sp. SBL4, which imposed no inhibitory effect on TYRO, all strains had moderate activity on this enzyme, at 10 mg/mL (Table 1), which indicates the presence of compounds in those species with potential interest for PD therapeutics [29].

**Table 1:** Acetylcholinesterase (AChE), butyrylcholinesterase (BuChE) and tyrosinase (TYRO) inhibitory activity (%) of methanol extracts of four strains belonging to the *Nannochloris* (SBL1 and SBL4), *Picochlorum* (SBL2) and *Desmochloris* (SBL3) genera. Values are represented as the mean and standard deviation (*n* = 6).

	AChE		BuChE		TYRO	
Species/Standard	1 mg/mL	10 mg/mL	1 mg/mL	10 mg/mL	1 mg/mL	10 mg/mL
SBL1	na	17.1 ± 5.7	52.0 ± 8.4	58.0 ± 7.4	22.7 ± 4.6	44.8 ± 5.1
SBL2	na	21.2 ± 8.1	66.1 ± 3.4	69.3 ± 2.5	32.6 ± 7.3	39.5 ± 5.4
SBL3	na	na	55.2 ± 6.5	60.4 ± 5.2	15.0 ± 5.2	40.1 ± 3.5
SBL4	na	na	59.0 ± 8.2	41.2 ± 12.0	10.6 ± 4.1	na
Galantamine *	93.2 ± 0.5	nt	80.3 ± 0.7	nt	-	-
Arbutin *	-	-	-	-	78.3 ± 0.1	nt

* Positive control; nt, not tested; na, no activity.

### 2.3. In Vitro Cytotoxic Activity

**Figure 2:**
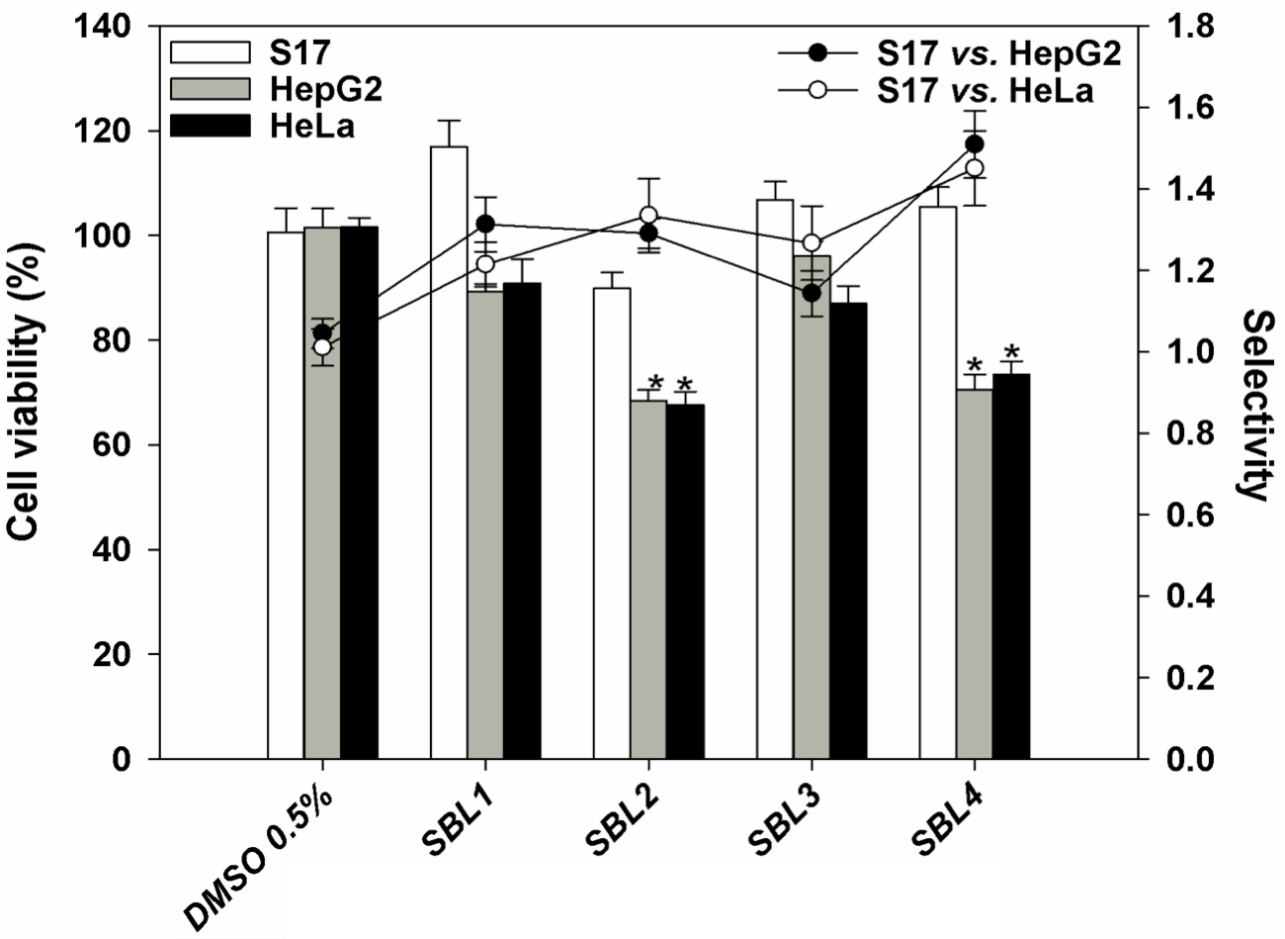
Effect of the application of methanol extracts of four strains belonging to the *Nannochloris* (SBL1 and SBL4), *Picochlorum* (SBL2) and *Desmochloris* (SBL3) genera, applied at a concentration of 125 µg/mL for 72 h, on the viability of human hepatocarcinoma (HepG2), cervical carcinoma (HeLa) and non-tumoral murine stromal (S17) cell lines, in comparison to a control without extract (DMSO, 0.5%, *v*/*v*). Bars and lines correspond, respectively, to cell viability and selectivity. * Significant differences (*p* < 0.001) compared with the control (*n* = 6). Half maximal inhibitory concentrations for etoposide used as a positive control were 1.9, 4.2 and 10 µg/mL for HepG2, HeLa and S17 cells, respectively.

According to the World Health Organization (WHO), cancer is responsible for about 13% (~7.6 million) of fatalities worldwide, being the second most common cause of death from disease after myocardial infarction. The current available antitumoral drugs generally display undesirable effects, making the search for more effective and safer drugs necessary. The algal extracts under study were thus tested against two human tumoral cell lines, namely: HepG2 (hepatocellular carcinoma) and HeLa (cervical carcinoma). Samples were applied for 72 h at a concentration of 125 µg/mL, and cell viability was assessed by the 3-(4,5-dimethylthiazol-2-yl)-2,5-diphenyltetrazolium bromide (MTT) colorimetric assay [30]. To evaluate selectivity, samples were applied to a murine non-tumoral cell line (S17, stromal cells). None of the extracts were cytotoxic to non-tumoral cells and displayed different degrees of toxicity towards tumoral cell lines (Figure 2). *Nannochloris* sp. SBL1 and *Desmochloris* sp. SBL3 did not reduce significantly the viability of any of the cell lines tested, suggesting a nontoxic nature for the compounds present in the extract of those species. Extracts from *Picochlorum* sp. SBL2 and *Nannochloris* sp. SBL4 significantly reduced the viability of both HepG2 and HeLa cells, with lower toxicity against non-tumoral S17 cells. However, *Nannochloris* sp. SBL4 extracts showed the highest selectivity index (SI), suggesting that this strain contains molecules with interesting antitumoral properties that may act selectively on cancer cells. Although microalgae have long been recognized as sources of important biomolecules with potential medical uses [31], there have been few reports on their cytotoxicity against human tumoral cells [32,33].

### 2.4. In Vitro Antileishmanial Activity

*Leishmania infantum* is the causative agent of canine leishmaniasis and both cutaneous and visceral forms of human leishmaniasis in the Mediterranean region [34]. The disease is endemic in all 22 countries of this area, where it is considered as a serious public health and veterinary problems [35]. In the Iberian Peninsula, the infection by *L. infantum* in humans is mainly related to immunosuppressive diseases, especially HIV co-infection [36,37]. Cases that are not the result of co-infection occur mostly in children. At present, there are no effective human or canine vaccines, and chemotherapy is the only means of controlling leishmaniases. However, currently applied drugs have high costs, depend on long-term administration and display high toxicity and reduced efficacy due to increasing parasite resistance [38,39]. Hence, the search for novel, safe, non-toxic and cost-effective drugs that can be used alone or in combination therapies to antileishmanial therapy and/or immunoprophylaxis is urgent [38,39,40,41].

**Figure 3:**
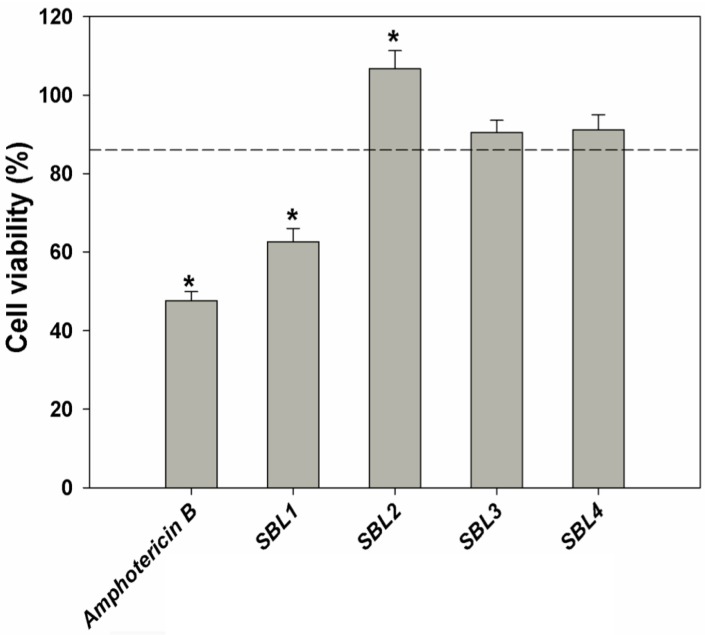
Effect of the application of methanol extracts of four strains belonging to the *Nannochloris* (SBL1 and SBL4), *Picochlorum* (SBL2) and *Desmochloris* (SBL3) genera at a concentration of 250 µg/mL for 48 h on the viability of *Leishmania infantum* promastigotes expressed as percentage (%), in comparison to a control (DMSO, 0.5%, *v*/*v*). Amphotericin B was used as the positive control. * Significant differences (*p* < 0.01) compared with the control (promastigotes treated with 0.5% DMSO, dashed line; *n* = 6).

In this work, samples were applied to *L. infantum* promastigotes at a concentration of 250 μg/mL for 48 h, and cell viability was determined by the MTT assay (Figure 3). *Nannochloris* sp. SBL1 was able to significantly reduce promastigotes viability down to 62%, as compared with untreated cells. Parasites treated with amphotericin B as the standard drug exhibited a viability of 47% at a concentration of 0.23 μg/mL (Figure 3). Marine organisms are recognized as a source of novel products and as a promising alternative to antileishmanial therapy and control [39,42]. However, there were no reports until now on the antileishmanial potential of microalgae. Assays are now being performed to ascertain the *in vitro* toxicity of this strain on intracellular amastigotes of *L. infantum.*

### 2.5. Extraction Yield and Phytochemical Analysis

The polarity and the nature of the solvents used in the extraction process define the extraction yield and the composition of the obtained extracts and, thus, their biological activity [43]. In this work, the dried biomass was extracted with methanol. Since it is known that this solvent has affinity for a broad diversity of bioactive compounds, namely phenolic compounds [44] and carotenoids, this approach already allowed the extraction of bioactive compounds from different species of microalgae [17]. The extraction yields were as follows: *Nannochloris* sp. SBL1, 29.9%; *Picochlorum* sp. SBL2, 32.2%; *Desmochloris* sp. SBL3, 33.9%; and *Nannochloris* sp. SBL4, 40.7%.

In spite of being considered as important sources of bioactive compounds, data on the phenolic content of microalgae is scarce [17,23,31,32,33,45,46], and to the best of our knowledge, there are no reports on the phenolic composition of *Nannochloris*, *Picochlorum* and *Desmochloris* microalgae. The accurate quantification of different phenolic structural groups remains difficult [47]. Spectrophotometric (colorimetric) techniques are widely used and are convenient when dealing with several samples of unknown composition [48]. The highest total phenolic content (TPC) was obtained in *Picochlorum* sp. SBL2 (114 mg gallic acid equivalents (GAE)/g DW), followed by *Nannochloris* sp. SBL4 (83.3 mg GAE/g DW) and *Desmochloris* sp. SBL3 (59.3 mg GAE/g DW). Those values are higher than those reported for methanol extracts of other species of microalgae, namely *Tetraselmis chuii*, *Nannochloropsis oculata*, *Chlorella minutissima* and *Rhodomonas salina* [17]. Only *Nannochloris* sp. SBL1 had a low content of phenolic compounds (5.8 mg GAE/g DW). Samples were further analyzed by reverse-phase HPLC, and three phenolic acids (gallic, coumaric and salicylic acids) were identified in *Nannochloris*, *Picochlorum* and *Desmochloris* microalgae samples (Table 2). The phenolic composition varied as a function of the strain analyzed, and *Picochlorum* sp. SBL2 had the highest sum of phenolic compounds (1.1 mg/g extract, DW), followed by *Nannochloris* sp. SBL4 (0.21 mg/g extract, DW), *Nannochloris* sp. SBL1 (0.12 mg/g extract, DW) and *Desmochloris* sp. SBL3 (0.07 mg/g extract, DW). Salicylic acid was the main phenolic detected in *Picochlorum* sp. (0.64 mg/g extract, DW) and at a lower level in *Nannochloris* sp. SBL4 (0.14 mg/g extract, DW). Coumaric acid was the only phenolic acid common to all strains and was detected in similar concentrations in *Nannochloris* (0.06 mg/g extract, DW) and *Desmochloris* strains (0.07 mg/g extract, DW).

Biophenolics are considered to be the most common secondary metabolites in photosynthetic organisms. These compounds are potent antioxidants due to their capacity to scavenge singlet oxygen and free radicals by donating hydrogen from the phenolic hydroxyl groups. This results in a stable end product that does not initiate or propagate lipid oxidation [49]. Such antioxidant ability confers phenolics an important role in the prevention of oxidative stress-related diseases, such as cancer and neurological disorders. However, the phenolic content of microalgae is lower than the levels reported for terrestrial plants [50]. One must keep in mind that, similarly to what was observed for other bioactive molecules, such as carotenoids, the phenolic levels in algal biomass can be increased through the modification of the growing conditions [51,52].

Microalgae are known producers of different carotenoids displaying potent antioxidant and anti-carcinogenic activities [53]. Therefore, the carotenoid composition of all extracts was determined by HPLC and is shown in Table 2. Neoxanthin (0.02–1.45 mg/g extract DW), violaxanthin (0.05–0.44 mg/g extract DW), lutein (0.19–1.29 mg/g extract DW), zeaxanthin (0.10–0.54 mg/g extract DW) and β-carotene (0.52–1.19 mg/g extract DW) were detected in all strains. Canthaxanthin was only detected in *Picochlorum* sp. SBL2 (1.15 mg/g extract DW). The pigment composition of *Picochlorum* sp. SBL2 extract is similar to that reported in a previous work [54], except for the presence of canthaxanthin. In fact, the strain of *Picochlorum* sp. studied by de la Vega and co-workers [54] was suggested by the authors as a promising feedstock for the exploitation of carotenoids and biodiesel production. Both *Nannochloris* strains had similar carotenoid composition. However, *Nannochloris* sp. SBL1 had a higher concentration of carotenoids than those detected in SBL4. Except for zeaxanthin, the identified pigments in *Nannochloris* strains were previously observed in *Nannochloris atomus* [55]. To the authors’ knowledge, the pigment composition of *Desmochloris* has never been reported previously. Regarding the total amount of carotenoids, *Picochlorum* sp. had the highest amounts (3.55 mg/g extract DW), followed by *Nannochloris* SBL1 (3.07 mg/g extract DW) and *Desmochloris* SBL3 (1.74 mg/g extract DW), whereas *Nannochloris* sp. SBL4 had the lowest content (1.62 mg/g extract DW).

**Table 2:** HPLC analysis of phenolic and carotenoid compounds (mg/g extract DW) of methanol extracts of four strains belonging to the *Nannochloris* (SBL1 and SBL4), *Picochlorum* (SBL2) and *Desmochloris* (SBL3) genera.

Compound	SBL1	SBL2	SBL3	SBL4
Gallic acid	0.06	0.11	nd	nd
Coumaric acid	0.06	0.35	0.07	0.07
Salicylic acid	nd	0.64	nd	0.14
**Total phenolics**	**0.12**	**1.1**	**0.07**	**0.21**
Neoxanthin	0.02	1.45	0.11	0.06
Violaxanthin	0.16	0.44	0.05	0.15
Lutein	1.29	0.89	0.60	0.19
Zeaxanthin	0.51	0.54	0.48	0.10
Canthaxanthin	nd	1.15	nd	nd
β-carotene	1.08	0.52	0.61	1.19
**Total carotenoids**	**3.07**	**3.55**	**1.74**	**1.62**

nd, not detected.

Although a direct relationship could not be established between either the phenolics or pigment composition and the RSA observed for the extracts, synergistic effects between different pigments are known to increase the RSA towards the DPPH radical [56]. In addition, interactions with or between phenolic compounds cannot be ruled out. Nonetheless, our results reveal that all strains of microalgae tested can be exploited as feedstocks for the production of carotenoids with high commercial value for different applications. There is increasing market demand for carotenoids with an estimated annual growth rate of 2.3% and a global market that can reach $1.4 billion by 2018 [57]. Carotenoids, such as lutein, have a wide applicability in nutritional and pharmaceutical/biomedical industries, because of their antioxidant and anti-carcinogenic properties [31], prevention of cognitive impairment [58], eye supplements formulation [54] and food and feed additives for the pigmentation industry [53].

These commercially-relevant metabolites (e.g., carotenoids and phenolics) can be key to the development and sustainability of a future microalgae-based biorefinery venture, coupling the exploitation of lipids for biodiesel and/or edible oils purposes with the co-production of fine and/or bulk chemicals with high commercial value for other biotechnological applications [59]. For example, carotenoid-containing streams coming from biomass down-processing, but unused for biodiesel production, could upgrade the total value of the feedstock and enable the future deployment of biorefineries for growing and processing microalgal strains already adapted to the conditions found in the Red Sea. Although these strains contain high-added value compounds, for the setup of a biorefinery, an effective downstream procedure enabling the separation of the oil/lipids from these streams, in laboratory and industrial settings, needs to be developed. Moreover, the development of a microalgae biorefinery must take into account the market size for these high-added value compounds. When lipids are used for the production of biofuels, the co-production of high value metabolites may saturate the market currently available for these niche products [60]. To overcome market-size limitations, extracted lipids found in the biomass can be diverted to applications other than biofuel production, including nutritional (food and feed), pharmaceutical and cosmetic industries. Alternatively, if biofuels must be produced for strategic reasons concerning fuel security, the generated co-products may be diversified, and novel emerging markets can be explored [60,61,62]. Some examples of possible emerging markets are active food packaging and organic aquaculture feed, which present increasing demands for components containing antioxidants, stabilizers and coloring agents from biological sources [63,64].

## 3. Experimental Section

### 3.1. Chemicals

All chemicals used in the experiments were of analytical grade. AChE (EC.3.1.1.7) from electric eel, BuChE (EC.232.579.2) from equine serum, acetylthiocholine iodide (ATChI), butyrylthiocholine iodide (BTChI), 5,5-dithiobis-(2-nitrobenzoic acid (DTNB), galantamine, gallic acid, Tween 40, pyrocatechol violet, DPPH and all commercial standards for HPLC were purchased from Sigma (Steinheim, Germany). Ethylenediamine tetraacetic acid (EDTA) and sodium carbonate (Na_2_CO_3_) were acquired from Fluka (Steinheim, Germany). Merck (Darmstadt, Germany) supplied ferrozine, copper sulfate pentahydrate and Folin-Ciocalteu (F-C), while methanol was obtained from Fischer Scientific (Loughborough, UK). Additional reagents and solvents were purchased from VWR International (Leuven, Belgium).

### 3.2. Microalgae Culture

Microalgae strains, namely *Nannochloris* sp. SBL1, *Picochlorum* sp. SBL2, *Desmochloris* sp. SBL3 and *Nannochloris* sp. SBL4 were previously isolated by Pereira *et al.* [9]. Biomass was cultured in agar plates (1.5% agar) and further grown on liquid medium in 80-mL test tubes using Guillard’s F/2 medium and enriched seawater, as described in Pereira *et al.* [65]. Cultures were grown for 12 days at 23 °C, at a photon flux density of 100 μmol·m^−2^·s^−1^ with a 24-h light photoperiod. Upon culturing, biomass was harvested by centrifugation (5000× *g*, 5 min) and freeze-dried until the extraction procedure.

### 3.3. Extraction

Dried microalgae biomass was mixed with methanol (1:40, *w*/*v*) and the cells disrupted using an IKA T10B Ultra-Turrax disperser for 2 min, on ice. Extractions were performed overnight at room temperature (RT, 20 °C) under continuous stirring. The supernatants were recovered from extracted biomass by centrifugation (10,000× *g*, 10 min), filtered (Whatman No. 4 filter) and dried on a rotary evaporator (45 °C) under vacuum. Dried extracts were resuspended in methanol to a final concentration of 20 mg/mL and stored at −20 °C.

### 3.4. Antioxidant Activity

#### 3.4.1. RSA on DPPH Radical

RSA on the DPPH free radical was evaluated according to the method of Brand-Williams adapted to a 96-well microplate scale [66]. The absorbance was measured at 515 nm in a microplate reader, and the RSA was expressed as percent inhibition, relative to a negative control, containing methanol in place of the sample. Butylated hydroxytoluene (BHT, 1 mg/mL) was used as a positive control.

#### 3.4.2. Metal Chelating Activity on Iron and Copper Ions

Iron chelating activity was determined by measuring the formation of the Fe^2+^-ferrozine complex according to Megías *et al.* [67], with some modifications. The change in color was measured in a microplate reader at 562 nm. Copper chelating activity was determined using pyrocatechol violet (PV), as described by Megías *et al.* [67]. The change in color of the solution was measured at 632 nm. The synthetic metal chelator EDTA was used as a positive control at the concentration of 1 mg/mL for both metals.

### 3.5. AChE and BuChE Inhibitory Activity

AChE and BuChE inhibitory activities were measured by the Ellman method [68] as described by Orhan *et al.* [69]. Briefly, 20 µL of each extract (1 and 10 mg/mL) were mixed with 140 µL of 0.1 mM sodium phosphate buffer (pH 8.0) and 20 µL of AChE or BuChE solution (0.28 U/mL) in 96-well microplates and incubated at RT for 15 min. The reaction was initiated by adding 10 µL of ATChI or BTChI (4 mg/mL) together with 20 µL of DTNB (1.2 mg/mL). The hydrolysis of ATChI or BTChI was monitored by the formation of the yellow 5-thio-2-nitrobenzoate anion as a result of the reaction of DTNB with thiocholines catalyzed by the enzyme, at 412 nm, using a microplate reader. Results were expressed as AChE and BuChE percentage inhibition relative to a negative control, containing methanol in place of the sample. Galantamine was used as the positive control (1 mg/mL).

### 3.6. TYRO Inhibitory Activity

The inhibitory activity against TYRO was determined by the method reported by Nerya *et al.* [70] with modifications, using l-tyrosine as the substrate. Samples (70 µL at the concentrations of 1, 5 and 10 mg/mL) were mixed in 96-well microplates with 30 µL of TYRO (333 Units/mL in phosphate buffer, pH 6.5) and incubated for 5 min. Then, 110 µL of substrate (l-tyrosine, 2 mM in water) were added to each well and further incubated for 30 min at RT. The optical densities of the wells were read at 492 nm. Results were expressed as TYRO percentage inhibition relative to a negative control, containing methanol in place of the sample. Arbutin was used as the positive control at the concentration of 1 mg/mL.

### 3.7. In Vitro Cytotoxic Activity

HepG2, HeLa and S17 cells were kindly provided by the Centre for Biomedical Research (CBMR), University of Algarve. The HepG2 cell line was maintained in RPMI-1640 culture media supplemented with glucose (1000 mg/mL), 10% heat-inactivated fetal bovine serum (FBS), l-glutamine (2 mM), penicillin (50 U/mL) and streptomycin (50 µg/mL). S17 and HeLa cells were grown in DMEM culture media supplemented with glucose (1000 mg/mL), 10% FBS, l-glutamine (2 mM), penicillin (50 U/mL) and streptomycin (50 µg/mL). Both lines were grown at 37 °C and 5.0% CO_2_ in a humidified atmosphere.

Exponentially-growing cells were seeded at a density of 5 × 10^3^ cells/well on 96-well plates and incubated for 24 h at 37 °C in 5.0% CO_2_. Then, the extracts (100 µL) were applied at a concentration of 125 µg mL for 72 h. Positive control cells were treated with etoposide at the same concentration and incubation period as the extracts, while negative control cells were treated with DMSO at the highest concentration used in test wells (0.5%, *v*/*v*). The MTT assay [30] was used to assess the effect of the extracts on mitochondrial metabolic activity, as an indicator of cell viability. Results were expressed in terms of cell viability (%).

### 3.8. In Vitro Antileishmanial Activity

Promastigote forms of *L. infantum* (MHOM/PT/88/IMT-151) were provided by the Medical Parasitology Unit of the Institute of Hygiene and Tropical Medicine (New University of Lisbon, Portugal) and maintained in RPMI-1640 medium supplemented with 10% heat-inactivated FBS, l-glutamine (2 mM), penicillin (50 U/L) and streptomycin (0.05 mg/L), at 24 °C in tissue flasks. For the determination of the antileishmanial activity, *L. infantum* promastigotes (1 × 10^7^ parasites/mL) were incubated in 96-well plates with the extracts at the concentration of 250 μg/mL, for 48 h. Positive control parasites were treated with amphotericin B at a concentration of 0.23 μg/mL and during the same incubation period as the extracts, while negative control cells were treated with DMSO at the highest concentration used in test wells (0.5% *v*/*v*). The MTT assay [30] was used to assess the effect of the extracts on parasites viability. After incubation, 20 μL of MTT (5 mg/mL in PBS) were added to each well, and plates were re-incubated for 2 h, at 37 °C. Then, plates were centrifuged (15 min, 4 °C, 1479× *g*), the supernatants discarded and 150 μL of DMSO added to each well in order to dissolve the formazan crystals. Absorbance was measured at 590 nm, and results were expressed in terms of cell viability (%).

### 3.9. TPC

The TPC of the extracts was determined by the F-C assay according to Velioglu *et al.* [71]. The extracts (5 µL at the concentration of 10 mg/mL) were mixed with 10-fold diluted F-C reagent in distilled water (100 µL) and incubated at RT for 5 min. Then, 100 µL of sodium carbonate (Na_2_CO_3_, 75 g/L, *w*/*v*) were added; samples were incubated for 90 min at RT, and the absorbance was measured at 725 nm, on a microplate reader. Results were expressed as GAE using a calibration curve of gallic acid standard solutions, in milligrams per gram of extract (mg GAE/g DW).

### 3.10. HPLC Analysis

#### 3.10.1. Analysis of Phenolic Compounds

The extracts at the concentration of 10 mg/mL in ultrapure water were analyzed by HPLC-DAD (Agilent 1100 Series LC system, Boeblingen, Germany) formed by the following modules: vacuum degasser (G1322A), quaternary pump (G1311A), autosampler (G1313A), thermostated column compartment (G1316A) and a diode array detector (G1315B). Data acquisition and instrumental control were performed by the software LC3D ChemStation (Version Rev.A.10.02(1757), Agilent Technologies, Boeblingen, Germany). Analyses were performed on a Mediterranea Sea18 column, 15 × 0.21 cm, 5-µm particle size (Teknokroma, Barcelona, Spain). The mobile phase consisted of a mixture of methanol (Solvent A) and 2.5% acetic acid aqueous solution with the following gradient: 0–5 min: 10% A, 5–10 min: 10%–30% A, 10–40 min: 30%–90% A, 40–45 min: 90% A, 45–55 min: 90%–10% A and 55–60 min: 10% A, using a flow of 0.5 mL/min. The injection volume was 20 μL with a draw speed of 200 μL/min. The detector was set at 210, 280 (used for quantification), 320 and 350 nm. For chemical identification, the retention parameters of each assay were compared with the standard controls and the peak purity with the UV-visible spectral reference data. The levels of the different compounds were extrapolated from calibration standard curves. Commercial standards (gallic acid, p-hydroxybenzoic acid, catechin, vanillic acid, caffeic acid, syringic acid, epigallocatechin gallate, coumaric acid, salicylic acid, ferulic acid, rosmarinic acid, 4-hydroxybenzaldehyde, apigenin, BHT, chlorogenic acid, epicatechin, epigallocatechin, flavone, gentisic acid, m-hydroxybenzoic acid, oleanolic acid, quercetin, resveratrol, rutin hydrate, trans-cinnamic acid and uvaol) were prepared in methanol (10,000 mg/L) and diluted with ultrapure water in the desired concentration.

#### 3.10.2. Analysis of Pigment Composition

All methanolic extracts were injected at the concentration of 10 mg/mL with an injection volume of 20 μL. Carotenoids were analyzed with a Knauer smartline 5000 HPLC equipped with a Knauer Smartline pump 1000 and Knauer UV detector 2600. The HPLC was performed using Luna 5u C18 100A (5 µm, 250 × 4.6 mm). The mobile phase consisted of acetonitrile (ACN) and ethyl acetate (EA) with the following gradient: 0–15 min: 100% ACN, 15–35 min: 50%–50% ACN, 35–45 min: 100% ACN, using a flow of 1 mL/min. The identification of compounds was achieved by comparing the retention time and the UV spectra with those of pure commercial standards. Quantification was performed using calibration curves prepared for each of the pigments analyzed (fucoxanthin, lutein, violaxanthin, neoxanthin zeaxanthin astaxanthin, canthaxanthin, chlorophyll *a*, lycopene and β-carotene).

### 3.11. Statistical Analysis

Results were expressed as the mean ± standard deviation, and experiments were conducted at least in triplicate. Significant differences were assessed by analysis of variance (ANOVA) or the Duncan’s new multiple range test when the parametricity of data did not prevail. SPSS statistical package for Windows (Release 15.0, SPSS Inc., Chicago, IL, USA) was used.

## 4. Conclusions

Our results indicate that biomass from the microalgae *Nannochloris* sp., *Picochlorum* sp. and *Desmochloris* sp. isolated from the Red Sea not only have fatty acid methyl ester profiles considered as ideal for biodiesel production [9], but also contain molecules with relevant bioactivities, including antioxidant, inhibition of BuChE and TYRO, cytotoxic and antileishmanial activities. Moreover, chemical characterization of the extracts of all strains revealed the presence of different phenolic and carotenoid compounds, some of which have high market value. Taken as a whole, these results suggest that the biomass of these microalgae is promising as feedstocks for supplying high-value compounds to the biomedical/pharmaceutical and food/feed industries. Future research will focus on the development of a suitable downstream procedure for the coupled extraction of lipids and target co-products studied in the present report.
